# A new genus of fossil Mymaridae (Hymenoptera) from Cretaceous amber and key to Cretaceous mymarid genera

**DOI:** 10.3897/zookeys.130.1241

**Published:** 2011-09-24

**Authors:** George Poinar Jr., John T. Huber

**Affiliations:** 1 Department of Zoology, 3029 Cordley Hall, Oregon State University, Corvallis, OR, USA, 97331–2914; 2 Natural Resources Canada, c/o AAFC, K.W. Neatby Building, 960 Carling Ave., Ottawa, ON, K1A 0C6, Canada

**Keywords:** Carpenteriana, Enneagmus, Macalpinia, Myanmymar, Triadomerus, generic key

## Abstract

*Myanmymar aresconoides* **gen n., sp. n.** is described from one female in Burmese amber, dated as about 100 my. It is similar to *Arescon* on wing features but is unique among Mymaridae in having distinctly segmented palpi. It is the fifth mymarid genus definitely referable to the Cretaceous period. A key to Cretaceous mymarid genera is presented and the features of *Myanmymar* are compared with the other Cretaceous and extant mymarid genera.

## Introduction

Members of Mymaridae, commonly called fairyflies, are relatively well represented in amber inclusions from the late Cretaceous (70–100 my) to the Miocene (20–40 my), and also in copal (Ross et al. 2010). Yoshimoto (1975) described four genera, *Carpenteriana*, *Macalpinia*, *Protooctonus* and *Triadomerus* in Mymaridae from Cretaceous amber inclusions originating near Medicine Hat, Alberta, and Cedar Lake, Manitoba, Canada. Gibson et al. (2007) transferred *Protooctonus* Yoshimoto to Mymarommatidae (Mymarommatoidea) and synonymized it under *Archaeromma* Yoshimoto, 1975. In addition to the mymarids, Yoshimoto (1975) also described three new genera in the family Tetracampidae and one new genus, *Enneagmus*, in the family Trichogrammatidae (Chalcidoidea). Huber (2005) transferred *Enneagmus* to Mymaridae. Though their affinities are uncertain the Cretaceous fossils assigned to Tetracampidae by Yoshimoto (1975) likely do not belong to that family (Gumovsky and Perkovsky 2005; Heraty and Darling 2009). One other family, the Khutelchalcididae (Rasnitsyn et al. 2004), was described from an impression fossil from the earliest Cretaceous or latest Jurrassic period, but Gibson et al. (2007: 106) concluded it does not belong to the Chalcidoidea. Thus, the only extant family of Chalcidoidea definitely extending back to the Cretaceous is the Mymaridae.

The new genus described below is from Burmese amber, which is widely accepted as being at least 100 million years old (Ross et al. 2010), and thus is the oldest known fossil mymarid. The amber was obtained from a mine, first excavated in 2001, in the Hukawng Valley southwest of Maingkhwan in Kachin State (26º20'N, 96º36'E), Burma (Myanmar). This new amber site, known as the Noije Bum 2001 Summit Site, was assigned to the Early Cretaceous, Upper Albian, on the basis of paleontological evidence (Cruickshank and Ko 2003), placing the age at 97–110 mya. Nuclear magnetic resonance spectra and the presence of araucaroid wood fibers in amber samples from the Noije Bum 2001 Summit site indicate an araucarian (possibly *Agathis*) tree source for the amber (Poinar et al. 2007). No Mymaridae have yet been described from even older amber, from Lebanon, which is dated as at least 121mya.

## Methods

The amber specimens were immersed in mineral oil Johnson’s baby oil) for photography. Photographs were produced with a high-resolution ProgRes C14+ camera mounted on a Nikon SMZ1500 stereomicroscope. Body measurements of the holotype are in micrometers and are approximate because they were taken from the printed and enlarged digital photographs. One abbreviation is used: fl_x_ = flagellar segment x.

## Taxonomy

### Myanmymar

Huber gen. n.

urn:lsid:zoobank.org:act:C6087128-0B8F-4FE0-972E-25F6AE0C7CF9

http://species-id.net/wiki/Myanmymar_Huber

Figs 1–3

#### Derivation of generic name.

The genus name is a euphonious combination of letters. The gender is neuter.

#### Generic diagnosis.

**Figures 1–3.**
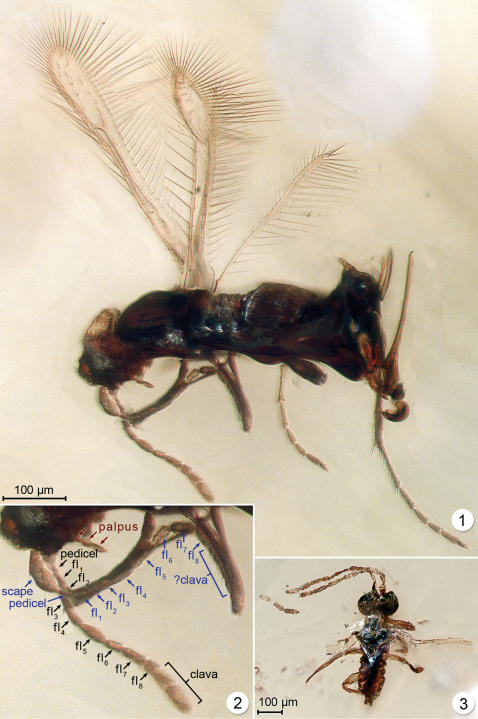
**1** *Myanmymar aresconoides*, female holotype **1** dorsolateral habitus **2** *Myanmymar aresconoides,* antennae and palpus **3** male of uncertain species identity, dorsolateral habitus.

Fore wing narrow, venation extending about two-thirds wing length, marginal vein probably longer than submarginal vein (wing base not clearly visible), and postmarginal vein apparently absent (Fig. 1); antenna with 8-segmented funicle and 2-segmented clava (Fig. 2); palpi (probably maxillary) distinctly 3-segmented (Fig. 2); tarsi 5-segmented and long; metanotum with anterior and posterior margins parallel; petiole ring-like, apparently shorter than wide; and ovipositor sheaths with several setae near apex.

#### Remarks.

**Figure 4.**
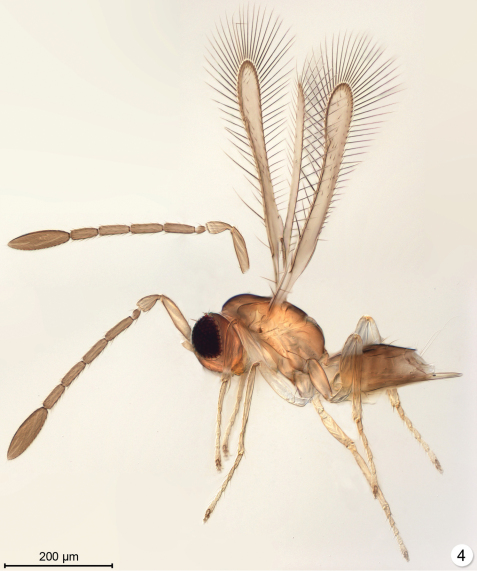
*Arescon* sp., an extant species from Thailand (lighting from below).

Among extant genera the closest in general appearance to *Myanmymar* are some females of *Arescon* Walker. Although very similar in wing shape and venation, *Arescon* females, exemplified by an unidentified extant species from Thailand (Fig. 4), differ from *Myanmymar* in having the funicle 5-segmented, clava 1-segmented, palpi 1-segmented, each ovipositor sheath with only one seta, and tarsi short.

*Myanmymar* exhibits three plesiomorphic features of Mymaridae: tarsi 5-segemented, female antenna with funicle 8-segmented, and fore wing venation longer than half wing length. Only three extant genera (*Boudiennyia*, *Eustochomorpha*, *Borneomymar*) share all these features; two others (*Gonatocerus*, *Ooctonus)* share the first two features but the venation is less than half the wing length (Huber 2002). *Myanmymar* is unique among described extant Mymaridae, and perhaps also the extinct genera, in having palpi with three distinct segments. The palpi are not visible in known representatives of the other extinct genera so that segmentation cannot be determined, but the fact that the palpi are not visible suggests that they are reduced, as in extant Mymaridae. The latter all have unsegmented palpi, with the single segment terminating in a long apical seta and one or two shorter, preapical setae.

#### Type species.

*Myanmymar aresconoides* Huber, sp. n.

### Myanmymar aresconoides

Huber sp. n.

urn:lsid:zoobank.org:act:0F33D265-8D23-4A52-B739-2235B6981CB8

http://species-id.net/wiki/Myanmymar_aresconoides

Figs 1–2

#### Holotype.

Female in amber inclusion in plastic box labeled (inside): “*Myanmymar aresconoides* ♀ Huber Holotype [red label]”, deposited in the Poinar amber collection maintained at Oregon State University.

#### Other material.

One male (OSUC) possibly belonging to *Myanmymar* was examined. It is in a triangular amber inclusion in same box as the holotype. It is excluded from type series because it is in poor condition (Fig. 3).

#### Description.

**Female.** Body length 535. Colour dark brown except antenna mostly, tarsi, ovipositor and wing venation brown and funicle segment 8 and clava lighter in colour (Fig. 1). *Head*. Width 120. Eye moderate in size, apparently with a few setae (Figs 1, 2). Gena wide, distinct. Maxillary palpi clearly 3-segmented (Figs 1, 2).

*Antenna*. Funicle segments 3, 5 and 7 distinctly longer than the remainder, and segments 3 and 5 distinctly the widest. Clava with apical segment slightly longer than basal segment. Measurements (length/width) taken from either left or right antenna: scape -/-, pedicel -/-, fl_1_15/15, fl_2_ 28/13, fl_3_ 30/13, fl_4_ 38/13, fl_5_ 38/13, fl_6_ 28/10, fl_7_ 28/15, fl_8_ 25/15, clava (total) 60/18.

*Mesosoma*. Length 200. Mesoscutum 100, about 1.6 times as long as scutellum (line of demarcation between the two not clear.) Metanotum 23, about ¾ as long as propodeum. Propodeum 30, about 1.2 times as long as metanotum.

*Wings*. Fore wing narrow, almost parallel-sided (about twice as wide near apex as at narrowest point), the posterior margin with a distinct, rounded lobe at level of base of marginal vein, and longest marginal setae much longer than wing width (Fig. 1). Wing surface without microtrichia, except sparsely in two fairly distinct rows beyond venation. Venation extending well beyond middle of wing. Marginal vein with about 8 setae along its length. Fore wing length/width 406/59, longest marginal setae 112, about 2 times greatest wing width. Hind wing (Fig. 1) narrow and parallel sided, its base not visible but presumably wing membrane not extending to base (as for most Mymaridae). Wing surface without microtrichia. Marginal setae at most about 7 times wing width.

*Legs*. Tarsi long, probably as long as tibiae (these not clearly visible), and metatarsomere 1 about 1.7 times as long as 2 (Fig. 1). Metatibial spur almost as long as metatarsal segment 3.

*Metasoma*. Length 225, longer than mesosoma. Petiole (not clearly visible) somewhat narrower than base of gaster. Gaster with segment 1 the longest (of those clearly demarcated). Ovipositor 218 (effective length); ovipositor sheaths slightly protruding beyond apex of metasoma, with several setae along apical third of exposed part (Fig. 1).

#### Derivation of species name.

After *Arescon*, the genus which *Myanmymar* most resembles based on the fore wing, and –oides, from Greek “eidos” meaning ‘resembling’ or ‘like’. The name is an adjective.

#### Remarks.

The specimen in Fig. 3 appears to be a male based on its antennal structure (probably 11-segmented, the number cannot be determined confidently) and apparent lack of an ovipositor. We tentatively associate this male with the female described as *Myanmymar aresconoides*, but it is impossible to be certain that it belongs to the same genus because it is so poorly preserved. Body length 445; head width 148.

#### Key to genera of Cretaceous Mymaridae. Females.

**Table:** 

1	Tarsi 3-segmented, funicle 5-segmented (not 4-segmented as stated in Yoshimoto 1975), and clava 3-segmented (Figs 5–8, and figs 22–24 in Yoshimoto 1975)	*Enneagmus* Yoshimoto
–	Tarsi 4- or 5-segmented; funicle 7- or 8-segmented	2
2(1)	Tarsi apparently 4-segmented (Figs 11, 12, and fig. 50A in Yoshimoto 1975) [funicle 8-segmented, clava 3-segmented]	*Macalpinia* Yoshimoto
–	Tarsi 5-segmented	3
3(2)	Funicle 7-segmented and clava 1-segmented (Figs 9, 10, and figs 18, 19 in Yoshimoto 1975)	*Carpenteriana* Yoshimoto
–	Funicle 8-segmented and clava 2- or 3-segmented	4
4(3)	Clava 3-segmented and wings wide, the marginal setae distinctly shorter than maximum wing width (Figs 13–16, and figs 13–17 in Yoshimoto 1975)	*Triadomerus* Yoshimoto
–	Clava 2-segmented and wings narrow, the marginal setae distinctly longer than maximum wing width (Figs 1, 2)	*Myanmymar* Huber

**Figures 5–8.**
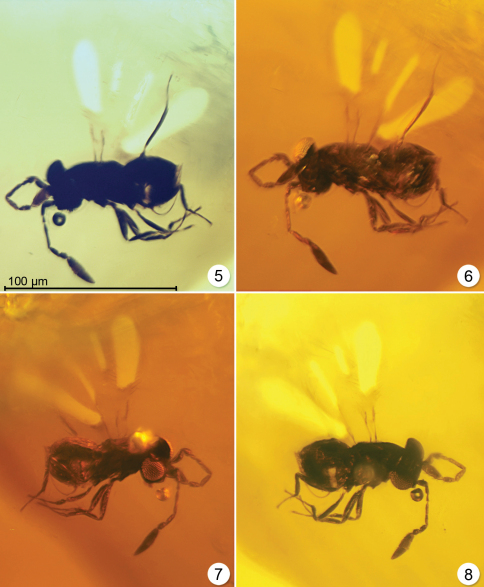
*Enneagmus pristinus*, holotype (left and right sides with lighting from below or the side).

**Figures 9–10.**
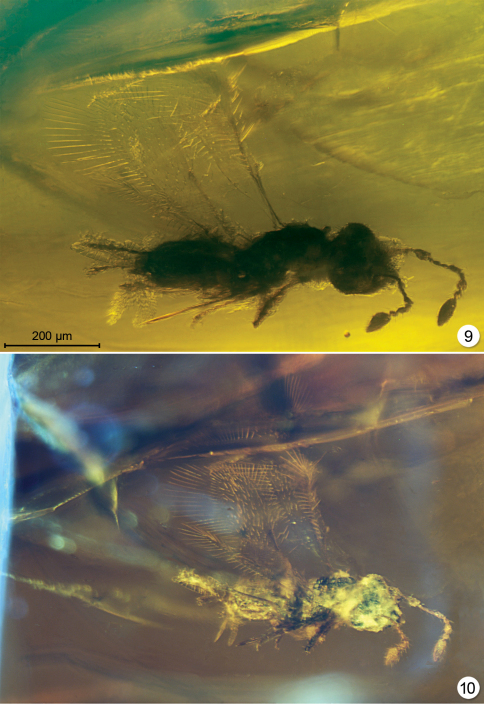
*Carpenteriana tumida*, paratype #5331 **9** lighting from below **10** lighting from below and diffused lighting from the side.

**Figures 11–12.**
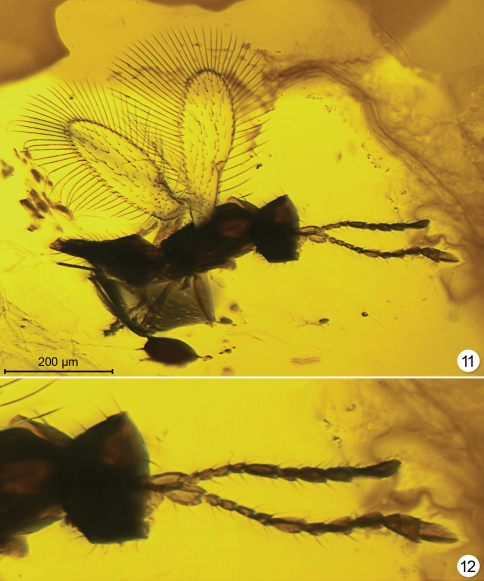
*Macalpinia canadensis*, holotype (lighting from below) **11** habitus **12** head and antennae.

**Figures 13–16.**
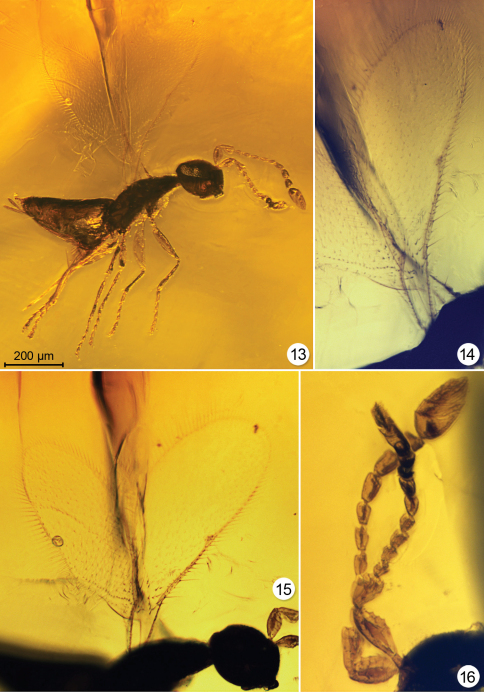
*Triadomerus bulbosus*, paratype # 5279 **13** habitus, lighting from the side and below **14** fore wing **15** wings **16** antenna (14–16 with lighting from below).

## Discussion

The late ichneumonid taxonomist Henry Townes once stated “If you don’t know what else to do, count”. For fossil mymarids his comment might better be worded “If you can’t do anything else, count and measure” because often not much else can be done with mymarid amber fossils except to count segments and measure their proportions. So no apology is needed for using almost exclusively the number of antennal and tarsal segments to distinguish genera. Townes’ quote actually applies quite well to fairyflies in general because the generic and family level classification of extant Mymaridae historically depended considerably on different combinations of antennal and tarsal segment numbers. Further refinements in defining family-group taxa within Mymaridae based on additional characters, e.g., petiole structure, gave fairly strong support to various proposed subfamilial classifications. At the genus-group level (or tribal level, if one chooses to recognize tribes) many other features besides segment number are, of course, required to define the taxa properly.

Four reasons are proposed here for the historical dependancy on segment numbers: 1) without good slide preparations, i.e., cleared, dissected and properly oriented (dorsal and lateral) specimens in a suitable permanent mounting medium, most body structures could not easily be examined or interpreted so they were often ignored, 2) meristic characters are unequivocal, so their use in identification keys (or to help define genera) make the keys relatively easy to use, 3) there is a considerable range in antennal and tarsal segment number among mymarid genera – among extant mymarids the number of funicle segments in females varies from 4–8, claval segments vary from 1–3, and tarsal segments of either sex from 3–5 (only one genus with 3) so different combinations can be used to help identify females of various genera; 4) the character is usually invariant within a mymarid genus – all or most of the species of a given genus will have only one combination of segment numbers. The wide range in, at least, number of tarsal segments in Mymaridae has evidently existed since the Cretaceous and is unexpected given that very few Cretaceous amber mymarid specimens have been found. Other families of Chalcidoidea, with few exceptions, are characterized by an invariant number of tarsal segments, either three, four or five. The number of tarsal segments in Mymaridae encompasses the range of all other chalcidoid families together and suggests that mymarids are either much older than the oldest fossils known (so they had lots of time to evolve the differences in segment number) or there was a sudden and rapid diversification just before the Cretaceous.

There is also considerable similarity among the Cretaceous genera. Three of them, but not *Carpenteriana*, have a 2- or 3- segmented clava. Four of them, but not *Enneagmus*, have a 7- or 8-segmented funicle. Only one extant genus, *Eustochomorpha*, also has 8 funicle segments in combination with 2 claval segments. The remaining 100+ extant genera have fewer segments, due to a seemingly independent reduction in number of either claval segments, funicle segments or both. Another similarity is the relatively long marginal vein in Cretaceous mymarids, except apparently in *Enneagmus*. Extant genera with a fore wing venation exceeding half the wing length are found mostly in the Southern Hemisphere, except *Arescon* which is more widespread. *Eustochomorpha* not only has a long marginal vein but also a long and well defined postmarginal vein, as in *Triadomerus*. The similarity of other features between Cretaceous and extant genera is also notable. *Triadomerus* appears to have a distinct, oblique hair line or asetose crease extending from the apex of the marginal vein to the posterior margin of the wing (Figs 13, 15), as in, e.g., *Australomymar*, *Boudiennyia*, and some *Ooctonus*. *Carpenteriana* and *Macalpinia* females have funicle segments that alternate in width (Figs 9, 11, 12) as in many extant species of Mymaridae, particularly some *Gonatocerus* (*Gahanopsis*) (illustrated in Triapitsyn et al. 2010). So although the tendency in evolution of Mymaridae appears to have been towards reductions in flagellar segment number and shortening of fore wing veins, Cretaceous genera do not appear very different from extant genera.

## Supplementary Material

XML Treatment for Myanmymar

XML Treatment for Myanmymar aresconoides
